# LILBID and nESI: Different Native Mass Spectrometry Techniques as Tools in Structural Biology

**DOI:** 10.1007/s13361-018-2061-4

**Published:** 2018-09-17

**Authors:** Oliver Peetz, Nils Hellwig, Erik Henrich, Julija Mezhyrova, Volker Dötsch, Frank Bernhard, Nina Morgner

**Affiliations:** 1 Institute of Physical and Theoretical Chemistry, J.W. Goethe-University, Frankfurt am Main, Germany; 2 Institute of Biophysical Chemistry, Centre for Biomolecular Magnetic Resonance, J.W. Goethe-University, Frankfurt am Main, Germany

**Keywords:** Native mass spectrometry, LILBID, nESI, Soluble proteins, Membrane proteins, Ion source

## Abstract

Native mass spectrometry is applied for the investigation of proteins and protein complexes worldwide. The challenge in native mass spectrometry is maintaining the features of the proteins of interest, such as oligomeric state, bound ligands, or the conformation of the protein complex, during transfer from solution to gas phase. This is an essential prerequisite to allow conclusions about the solution state protein complex, based on the gas phase measurements. Therefore, soft ionization techniques are required. Widely used for the analysis of protein complexes are nanoelectro spray ionization (nESI) mass spectrometers. A newer ionization method is laser induced liquid bead ion desorption (LILBID), which is based on the release of protein complexes from solution phase via infrared (IR) laser desorption. We use both methods in our lab, depending on the requirements of the biological system we are interested in. Here we benchmark the performance of our LILBID mass spectrometer in comparison to a nESI instrument, regarding sample conditions, buffer and additive tolerances, dissociation mechanism and applicability towards soluble and membrane protein complexes.

**Electronic supplementary material:**

The online version of this article (10.1007/s13361-018-2061-4) contains supplementary material, which is available to authorized users.

**Graphical Abstract:**
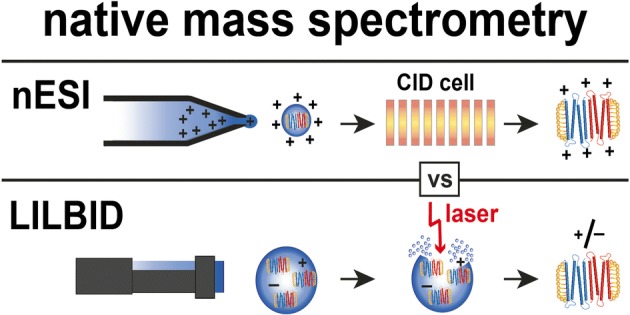
ᅟ

## Introduction

Native mass spectrometry (MS) has emerged as an important tool in structural biology [1, 2]. Advantages of MS compared to other tools like X-ray crystallography or nuclear magnetic resonance are for instance its lower limits of detection, its speed and its capability to deal with heterogeneous samples [3–5].

Electrospray ionization (ESI) and its variant nanoelectrospray ionization (nESI) followed by matrix-assisted laser desorption/ionization (MALDI) are the most prominent ion sources in MS worldwide. They reliably deliver valuable results for soluble proteins [6] but are not universally applicable for the more challenging matrices which are often required for membrane protein complexes.

Therefore, further development of MS instrumentation is still of high interest for the scientific community [7]. One promising newer method is laser-induced liquid bead ion desorption (LILBID), which employs a mid-Infrared (IR) laser to release ions from sample droplets of aqueous solution [8]. Like nESI, LILBID allows the analysis of proteins as well as larger intact protein complexes, as was shown for the example of an entire ATP synthase, consisting of 25 subunits in total. LILBID was able to reveal the complex intact or reveal constituting subunits and subcomplexes by increasing the desorption laser power. This dissociates the ATPase, resulting in mass spectra showing all of its subunits, as well as subcomplexes [9]. Similar experiments with nESI could show some ATPases intact and dissociation via collisional activation revealed some of the peripheral subunits [10]. This example shows how both methods can provide complimentary information on the quaternary structure of larger complexes.

In particular since last year, the scientific community is becoming aware of the LILBID technique [11–14] and some groups already implementing ion sources which are based on LILBID or quite close to it [15–17].

In our lab, we use the home-built LILBID mass spectrometer as well as a commercially acquired nESI instrument—a Synapt G2S from Waters. Both instruments have their advantages and disadvantages and are used depending on the requirements of the system under investigation. The aim of this work is to show pros and cons of both methods in more detail and to provide an overview on current limitations of both techniques for the investigation of intact biomolecular (membrane) protein complexes.

## Experimental

### Chemicals

TRIS [2-Amino-2-(hydroxymethyl)propan-1,3-diol], HEPES [2-(4-(2-Hydroxyethyl)-1-piperazinyl)-ethansulfonsäure], NaCl and KCl was purchased from Carl Roth (Karlsruhe, Germany) in the highest available purity.

Ammonium acetate in highest purity and the detergent OG [octyl β-*D*-glucopyranoside] were purchased from Sigma Aldrich (Darmstadt, Germany).

The detergent DDM [*n*-dodecyl-β-*D*-maltoside] was purchased from AppliChem (Darmstadt, Germany) or Anatrace/Affimetrix (Santa Clara, USA). DMPG [1,2-dimyristoyl-*sn*-glycero-3-phospho-(1′-*rac*-glycerol)] was purchased from Avanti Polar Lipids (Alabaster, USA) and glycerol (> 99%) was purchased from Alfa Aesar (Karlsruhe, Germany).

TPP [tetraphenylphosphonium chloride], Brij 35 [polyoxyethylene-(23)-lauryl-ether] and desthiobiotin were purchased from Sigma Aldrich (Darmstadt, Germany). TCEP [Tris-(2-carboxyethyl)-phosphin hydrochloride], sodium cholate, and imidazole were purchased from Carl Roth (Karlsruhe, Germany).

The Ni-NTA resin and the StrepII-Tactin resin were purchased from Qiagen (Hilden, Germany) and IBA (Goettingen, Germany), respectively.

### Sample Preparation

Lyophilized Avidin from Merck (Darmstadt, Germany) was dissolved in 3 M ammonium acetate buffer at pH 6.8 to substitute sodium counter ions to ammonium ions.

The membrane proteins EmrE [18, 19], DgkA [20], and KcsA [21, 22] were expressed via a continuous exchange cell-free system (CECF) utilizing lysates from *E. coli* A19 cells and based on T7-RNA polymerase transcription. T7-polymerase expression and purification, lysate preparation and CECF expression were performed as described previously [23, 24].

EmrE and DgkA were expressed in the precipitate forming mode (P-CF mode) as described in full detail elsewhere [23] and solubilized in DDM. DgkA was further expressed in the detergent mode (D-CF) [23] with 0.4% Brij 35 in the reaction as well as the feeding mixture.

To supply lipids for efficient complex formation, KcsA was expressed in the lipid (L-CF) mode in the presence of 20 μM nanodiscs assembled with DMPG and the scaffold protein MSP1E3D1 as described elsewhere [25].

### nESI and LILBID Mass Spectrometry

The nESI-MS instrument Synapt G2-S from Waters (Manchester, UK) upgraded with a 32-kDa quadrupole was operated in the positive nESI mode. Critical instrumental voltages and settings are displayed in the supplementary information.

LILBID-MS is an in-house-developed MS technique, which uses laser desorption for the release of sample ions from solution. A more detailed explanation follows in the next subchapter and the critical instrumental settings are displayed in the supplementary document.

Data analysis of nESI and LILBID was done using the software *UniDec* [26], *Mass*ign [27], and *OriginPro 2016*.

### LILBID-MS

**Figure 1:**
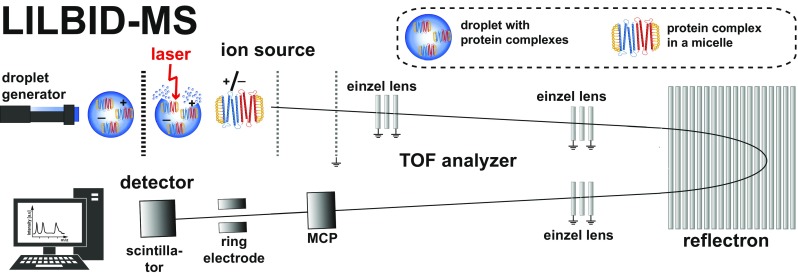
Schematic setup of the homebuilt technique LILBID-MS

A piezo-driven droplet generator (MD-K-130 from Microdrop Technologies GmbH, Norderstedt, Germany) is used to produce droplets of 50 μm diameter with a frequency of 10 Hz at 100 mbar. Samples are directly loaded into the droplet generator. The generated droplets are transferred to high vacuum and irradiated by an IR laser directly in the ion source, as indicated in Figure 1. The pulse length is 6 ns with a maximum energy of 23 mJ. The laser is a standard Nd:YAG laser and works at 10 Hz. The wavelength is tuned by an LiNbO_3_ optical parametric oscillator [28] to 2.94 μm ± 5 nm, the absorbing symmetric and asymmetric O–H stretching vibration of water. The laser power was measured by an optical power meter (PM100D, Thorlabs, Munich, Germany).

The droplet irradiation leads to an explosive expansion of the sample droplet and solvated ions are released and analyzed in a homebuilt time-of-flight setup including a reflectron, operating at 10^−6^ mbar [8].

The ion source is using ion optics based on a Wiley-McLaren type accelerator [29]. The ions enter the flight tube and are guided towards the detector via a reflectron.

The detector setup is based on a Daly-type detector [30]. Both ion modes can be used. For this work, ion detection was done in the negative mode. Spectra processing was done by using the software *Mass*ign [27], based on *LabVIEW*. The shown mass spectra are averaged signals of 500 droplets (measurement time of 50 s).

### Ion Sources nESI and LILBID

The most notable differences between nESI and LILBID are briefly explained in following.

**Figure 2:**
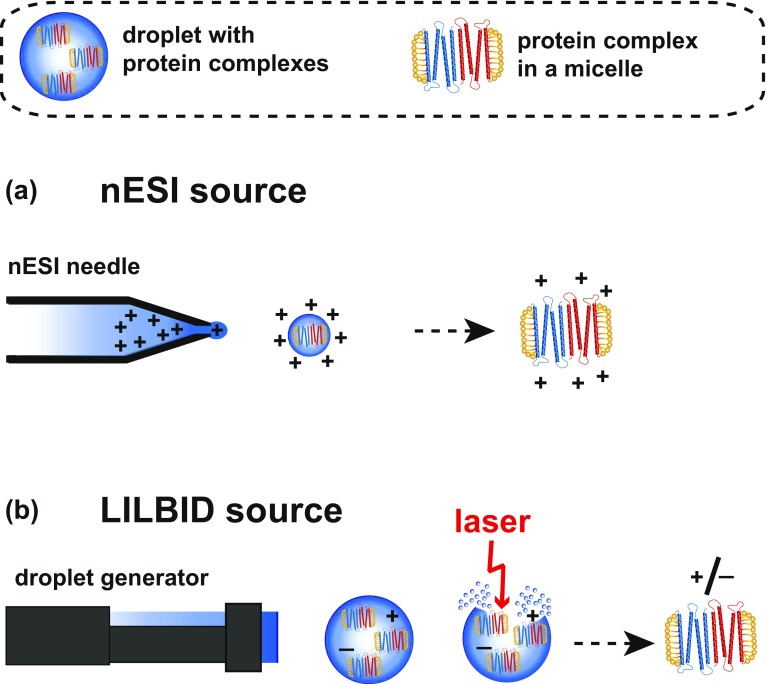
Ion sources for the example of a membrane protein complex: (**a**) a commercially available nESI-MS source, producing highly charged complexes; (**b**) the ion source of the homebuilt LILBID instrument, adding no additional charges to the solution net charge of the complexes

The charge of the protein complexes detected by nESI is determined by the polarity of the voltage applied to the nESI capillary. Briefly, in the nESI ion source a voltage is applied to the capillary loaded with the sample, which produces a spray of charged droplets. After evaporation and several droplet fissions, the highly charged protein complex remains (Figure 2a). ESI instruments are generally biased towards measurements in cationic mode, and, therefore, the majority of nESI studies is performed in this mode.

With LILBID, biomolecular complexes are not actively charged and therefore LILBID ions carry generally less charges than nESI ions, as indicated in Figure 2. As no active charging occurs in the LILBID process, the polarity of the charged protein ions reflects their solution net charge and depends on its isoelectric point and the pH of the buffer solution [31]. This means, anionic and cationic charged complexes can be released from the droplet at the same time, as indicated in Figure 2b. Both ion modes are routinely used for protein analysis.

### Ion Release in nESI and LILBID

**Figure 3:**
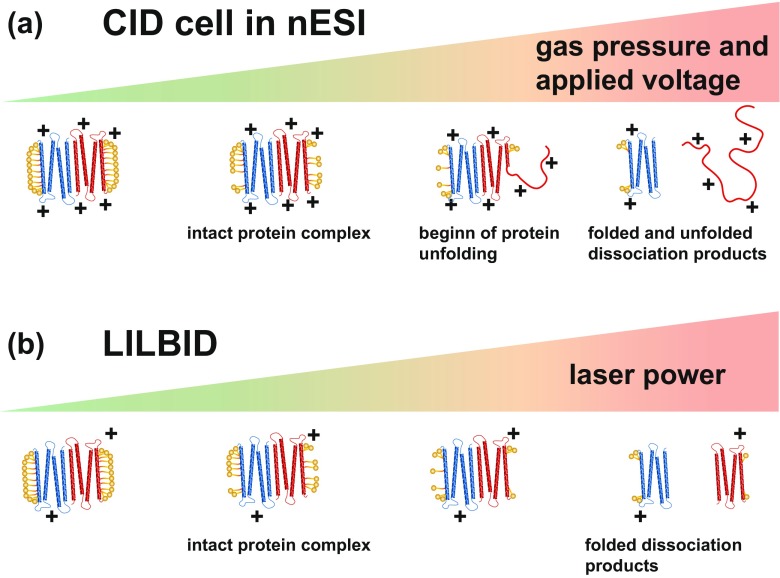
Basic work principle of a CID cell (**a**). Increasing gas pressure and applied voltage remove complex attachments and the complex itself dissociates via charge driven unfolding. In LILBID, (**b**) removal of attachments and degree of complex dissociation are controlled by the laser

nESI and LILBID both produce charged gas phase ions which can mostly be kept intact under soft instrument conditions. Instrumental parameters can be varied to achieve a soft transition of the ions into gas phase, to manipulate gas phase clean-up or to trigger complex dissociation. In nESI instruments, generally, a collision cell allows to remove unspecific attachments like salt and buffer molecules from the protein complex. The protein complex continuously collides here with inert gas molecules driven by a collision voltage inside the cell. Raising the collision voltage leads to increased removal of the complexes attachments. A competing process upon increased activation energy (collision voltage), is the charge driven unfolding of the protein, which results in dissociation of an unfolded monomer, as shown in Figure 3a. This event is termed collision-induced dissociation (CID) [32, 33].

For LILBID ions, the complex cleanup or complex dissociation can be controlled by the desorption laser. Increase of the laser power results in less attachments and dissociation of all of the proteins constituting the complex without prior unfolding (Figure 3b).

## Results

### Oligomeric State of Proteins

In the following, we compare the well-investigated water soluble protein complex Avidin and the membrane protein complex EmrE. The instrumental performance of both instruments was investigated using different sample conditions.

Avidin is a four-time biotin binding tetramer [34, 35] and EmrE is a dimeric multidrug efflux pump [18, 19]. Thirty micromolar EmrE complex (60 μM monomer) and 10 μM Avidin complex (40 μM monomer) are used for a comparative screening in different buffers, buffer concentrations and additives like salt to explore the current limitation of both MS techniques. If not indicated differently, the buffer used was 100 mM ammonium acetate, at pH 6.8 and the EmrE (membrane protein) samples contained additionally 5× critical micelle concentration (CMC) DDM.

**Figure 4:**
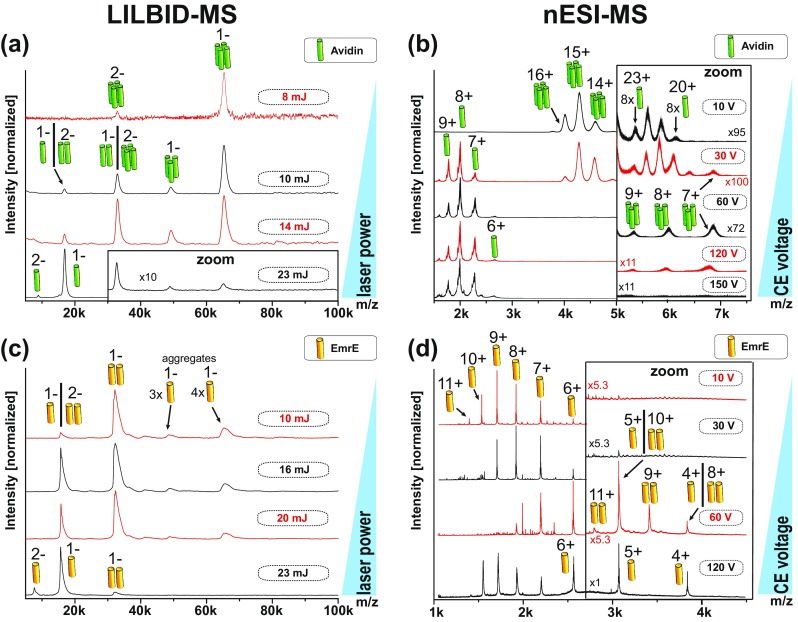
Different complex dissociations pathway of proteins investigated by LILBID-MS and nESI-MS. Laser power dependent dissociation of Avidin (**a**) and EmrE (**c**) using LILBID-MS. Collision-induced dissociation of Avidin (**b**) and EmrE (**d**) in nESI-MS shows a different dissociation pathway. The different oligomerization states are indicated by pictograms. For better visibility the insets show areas with small signals zoomed by the indicated factors

Figure 4 shows mass spectra of Avidin and EmrE taken with both instruments. Under soft instrumental conditions (low laser power: 8 mJ; and low activation energy: 10 V respectively) the dominating complex for Avidin is the expected tetramer in both cases (Figure 4a, b, top row spectrum). Under the same conditions, EmrE can be seen as a dimer with LILBID but appears monomeric with nESI (Figure 4c, d, top row spectrum). Increase of laser power or collision energy changes the character of all spectra.

Figure 4a shows LILBID spectra of the Avidin complex depending on the desorption laser power. The predominant tetrameric Avidin complex (at 10 mJ) undergoes increasing dissociation with increasing laser power from 10 to 23 mJ (top to bottom). At 14 mJ the Avidin dimer is present at similar intensities as the tetramer and at 23 mJ mainly monomer is visible.

For comparison, Figure 4b shows the Avidin complex at different instrumental conditions with nESI-MS. The degree of complex dissociation rises with increased activation. Increasing the collision voltage from 10 to 150 V, leads to dissociation of the Avidin tetramer into trimers via the release of unfolded monomers (from top to bottom). Almost the entire complex is dissociated into Avidin monomers at 150 V.

The LILBID spectra of the dimeric EmrE at different laser power settings are shown in Figure 4c. At low laser power of 10 mJ, EmrE dimer is the predominant species in the spectrum. With increasing laser power, more complex dissociates into monomers until almost the entire EmrE dimer is dissociated at 23 mJ. Small amounts of EmrE aggregates (trimer and tetramer) are also detectable up to 20 mJ laser power.

Figure 4d shows the EmrE complex investigated with nESI at different CID voltage settings. At 10 V and 30 V, only (partially) unfolded EmrE monomer can be observed with charge states 6+ to 11+. At least 60 V collisional activation is required to release intact EmrE dimer from the DDM micelle. Further increase of the collision voltage then causes complex dissociation into monomers, leading to a second charge distribution of folded monomers from 4+ to 6+. At 120 V, EmrE is completely dissociated into monomers.

These results show that both methods are soft ionization methods and reflect the correct oligomeric state of the water soluble Avidin at soft instrument settings.

The membrane protein complex EmrE is more challenging due to the detergent micelles, which need to be present to keep the complex soluble. The nESI process requires higher collision energies to strip the detergent molecules of the membrane complex to reveal the dimeric EmrE. The collision energies that are sufficient for the release of the protein complex are already above the threshold, which can cause complex dissociation. No instrumental conditions could be found showing the dimer of the membrane protein EmrE as the predominant species, which is in line with previous studies [36].

At low laser power of 10 mJ, LILBID shows almost exclusively EmrE dimer. No laser activation is needed to remove the detergent micelle, as the complex loses detergent in the same manner as water and buffer molecules in the laser desorption process. This shows that LILBID is especially suited to preserve intact membrane proteins.

An additional aspect that is noticeable is the occurrence of oligomeric states higher than the native ones.

A low amount of octameric Avidin is visible in nESI spectra taken at soft settings (Figure 4b, zoom in region at 10 V and 30 V). The occurrence of aggregates is already known from literature [37, 38]. These aggregates could not be found by LILBID at the 10 μM protein concentration (see Figure S1 for extended *m*/*z* range), despite both instruments showing similar limits of detection for all proteins (appendix Figure S1). At higher protein concentrations, aggregates can be found in LILBID as well. A more detailed investigation of the concentration dependent aggregates of proteins is shown in Figure S1 (Appendix). The differences can be explained by the droplet shrinking in the nESI process, which leads to an increase of the protein concentration in the spray droplets, which can cause aggregation. The explosive expansion of the LILBID process causes no concentration increase. The low intensity trimers and tetramer we see in the LILBID spectra of EmrE are therefore no result of a concentration effect. Instead, they are due to aggregation at the here-used solution conditions. Higher detergent concentrations remove the aggregates (Figure S5), which might be the reason why the increasing detergent concentration in the shrinking nESI droplets prevents occurrence of EmrE aggregates in the nESI spectra.

### Complex Dissociation Pathways

LILBID and nESI reveal the native oligomeric states of the proteins Avidin and EmrE but show completely different complex dissociation pathways for the tetrameric Avidin.

The nESI measurements in Figure 4b show Avidin dissociation in the asymmetric dissociation pathway typical for CID, producing exclusively trimers (starting at 30 up to 120 V) and unfolded monomers in the gas phase, which confirms previous findings on tetramer dissociation [37, 39, 40]. Voltages above 120 V lead to complete complex dissociation.

The dissociation process via CID is well known. Peripheral monomeric subunits of a protein complex [33] (consisting out of *n* monomers) dissociate via a charge driven unfolding process in the CID. The energy transfer from collisions is a slow multistep process occurs on a timescale of milliseconds [33]. During the unfolding process, the monomer takes up a high proportion of the initial complex charges and dissociates from the complex, which remains as accordingly lower charged complex (consisting of *n*-1 monomers) [33, 39, 41].

With LILBID, dissociation of the protein complex can be triggered by increased laser irradiation. For Avidin, this leads to a symmetric dissociation of the tetramer into dimers and at even higher laser intensities into monomers. No LILBID settings could be found, showing the trimer as more than a minor species, indicating the dissociation pathway into monomers via the dimeric state is predominant. These findings can be correlated to the quaternary structure of Avidin, showing Avidin tetramer is a dimer of dimers, with a much weaker binding interface between the two dimers [34].

The different energy transfer for both methods can explain these results. The LILBID desorption laser transfers enough energy on a short timescale to the investigated system, which redistributes through the protein. Other than the slow multistep CID process, this causes no (charge driven) rearrangement/unfolding and the dissociation occurs mainly along the weakest protein interfaces first, reflecting the different interaction strength between subunits.

LILBID can therewith provide information about the complex interfaces as well as next neighbor relationships. In contrast, collisional activation in nESI mass spectrometers can reveal information about peripheral positions of subunits. Both can be helpful especially for analyzing larger complexes. These results show that LILBID and nESI can lead to complementary structural information about the quaternary complex structure of interest.

### Buffer Tolerance of LILBID and nESI

Biological samples often require specific buffer conditions to mimic a cellular environment, essential for protein folding and activity. The buffer requirements can have a deteriorating effect on MS spectra quality.

**Figure 5:**
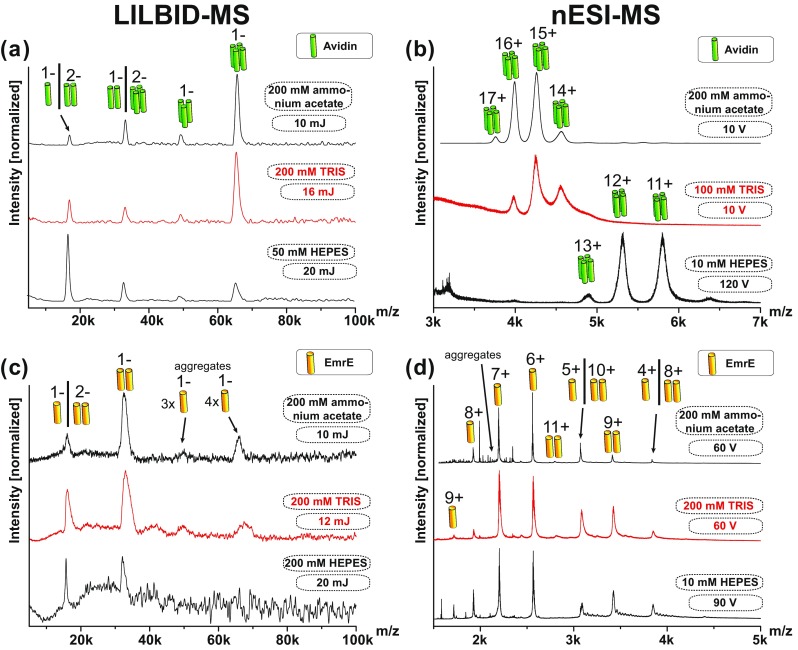
Different buffer tolerances of LILBID-MS and nESI-MS using Avidin and EmrE. Avidin tetramer and EmrE dimer in buffers containing up to 200 mM ammonium acetate were detectable in LILBID-MS (**a**,**c**) and nESI (**b**,**d**). TRIS (**a**,**b**) and HEPES buffer (bottom **a**–**d**) are worse to handle for both instruments, requiring increased laser power and CID voltage with increasing buffer concentrations as indicated. All EmrE buffers contain additionally 5× CMC DDM

Here we investigate the tolerance of nESI and LILBID for Avidin and EmrE towards different buffers. The effect of the buffers ammonium acetate (pH 6.8), TRIS (pH 7.5), and HEPES (pH 8.0) at concentration of 10 mM, 50 mM, 100 mM, and 200 mM (Appendix Figure S2 and Figure S3) was investigated. The EmrE buffer always included additionally 5× CMC DDM. The highest buffer concentrations of this buffer screening, which still allow the detection of the native protein complexes, are presented in Figure 5.

Up to 200 mM of the volatile buffer ammonium acetate, a preferred buffer for mass spectrometry, pose no challenge for both instruments.

As known for soluble proteins [42] and membrane proteins [43] increase of ammonium actetate buffer leads to improved mass resolution for the spectra taken with nESI, indicating the improved evaporation of the volatile buffer, removing other attachments at the same time. No such effect is observed in the LILBID spectra, as the laser desorption process is buffer independent.

Avidin complex in non-volatile buffer TRIS was clearly detectable in up to 100 mM TRIS with nESI and at 200 mM TRIS with LILBID. EmrE dimer could be found in up to 200 mM TRIS for both instruments (Figure 5c, d).

HEPES buffer is much more challenging for MS applications, showing detection limits for the protein complexes for nESI already at 10 mM HEPES for both proteins (Figure 5b, d). The limitation for the use of HEPES buffer with LILBID are 100 mM HEPES for the Avidin complex and around 200 mM HEPES for the EmrE complex. Both instruments needed harsher settings (higher CID voltage in nESI and higher laser power in LILBID) to release the complexes from HEPES buffer.

### Influence of Salt on LILBID and nESI Spectra

Like in cells, sodium and potassium ions can play a crucial role in functionality or even stability of proteins but have a negative effect on MS resolution. We investigate the effect of different salt concentrations for Avidin and EmrE on both MS instruments.

Appendix Figure S4 shows resolved LILBID spectra of Avidin tetramers up to 100 mM NaCl (Figure S4a) and 100 mM KCl (Figure S4c). Between 40 mM and 100 mM salt (NaCl or KCl) peak broadening and loss of intensity becomes significant due to increasing salt attachments to the complex. Due to the comparably low charges LILBID ions carry, the signal intensity at 120 μM NaCl or 100 μM KCl would still be sufficient to determine the charge state unambiguously and therewith the oligomeric state of the complex.

Increasing salt attachments with higher salt concentration shift the apparent mass of the complex to higher *m*/*z* ratios, as indicated by dotted lines, shown for LILBID and nESI (Figure S4a–d).

With nESI the tetrameric Avidin complex can be revealed for 40 mM NaCl or 40 mM KCl despite a significant loss of intensity between 10 mM and 40 mM salt (Figure S4b, d).

Figure S5a, b shows the influence on the spectra quality of the EmrE dimer with increasing NaCl concentration. The oligomeric state of EmrE dimer can be obtained in up to 40 mM NaCl in LILBID (Figure S5a) and 20 mM NaCl in nESI (Figure S5b).

### Influence of Glycerol on LILBID and nESI

To prevent freezing/thawing damage of proteins which are frozen for storage, stabilizing agents are often added before freezing. Up to 20% glycerol is used to prolong the storage time of frozen protein samples in biochemical labs. Therefore, the influence of glycerol on the mass spectra for Avidin tetramer and EmrE dimer was under investigation.

The intact complexes can be detected in buffer containing up to 20% glycerol (Figure S4e and Figure S5c) with LILBID. The limit for nESI is between 5 and 10% glycerol (Figure S4f and Figure S5d), above which EmrE is still visible, but only as a monomer.

Two effects can be observed with both instruments after adding glycerol to the protein complexes Avidin and EmrE.

Firstly, suppression of complex signals increases with rising glycerol concentration. Differently to salt, glycerol has no effect on the observed *m*/*z* values for the protein complexes, indicating that no molecules stay as adducts on the protein complexes in MS spectra.

Secondly, glycerol affects the observed oligomerization states in favor of the smaller complexes. Figure S4e shows the dissociation of the Avidin tetramer into monomers with increasing glycerol in LILBID. Between 5 and 10% glycerol, Avidin tetramer is no longer the predominant species in LILBID (Figure S4e). The same effect can be observed in nESI at 3% glycerol (Figure S4f). This general trend is also observed for EmrE (Figure S5c, d). This finding is an instrument-independent result and glycerol removal should be considered if investigating oligomers with MS after freezing with glycerol.

Noticeable is a shift in charge distribution at 3% glycerol for the Avidin complex in nESI spectra (Figure S4f) which can be explained by the supercharging effect of glycerol. Reduced protein stability with increasing glycerol amount and its supercharging effect in nESI has already been reported [44]. No supercharging effect of glycerol was observed in LILBID spectra.

### Influence of Detergent Concentration

Due to the hydrophobic nature of membrane proteins amphipathic agents like detergents are employed to avoid membrane protein aggregation or precipitation. Detergents are often used in higher concentrations, sometimes up to 20× CMC, which can have a hampering effect on many methods for structural biology. We analyzed EmrE in a detergent concentration series ranging from 3× CMC DDM up to 20× CMC DDM to investigate how the detergent concentration might influence LILBID and nESI spectra.

Figure S5e, f show no significant influence on the spectral quality of EmrE from LILBID or nESI with increasing DDM concentration up to 20× CMC.

Interestingly, LILBID spectra reveal reduction of the unspecific EmrE aggregates of trimer and tetramer with increasing amount of DDM (Figure S5e).

### Higher Oligomeric Membrane Proteins

In order to obtain a broader view regarding membrane proteins, we chose two additional membrane proteins for investigation. The quaternary structures of both proteins are known and they have an oligomeric state higher than EmrE: KcsA is a tetrameric potassium channel [21, 22] and DgkA is a trimeric enzyme [45], responsible for the conversion of diacylglycerol to phosphatidic acid [20].

**Figure 6:**
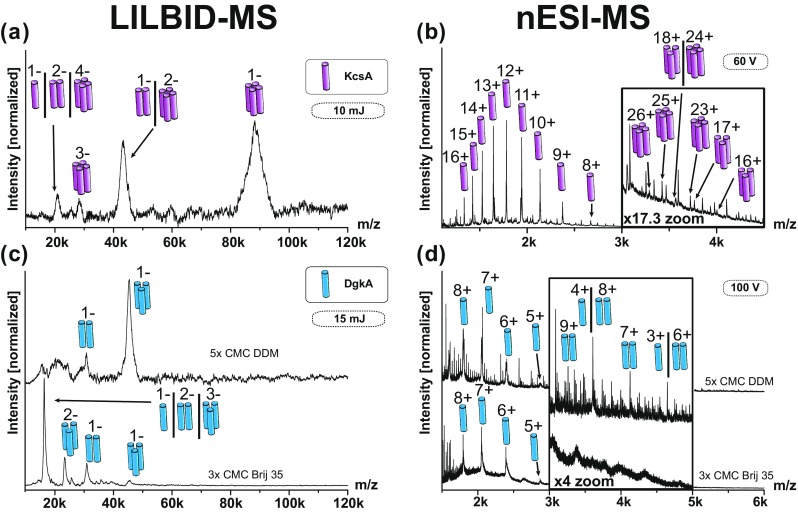
Different membrane proteins investigated with LILBID-MS and nESI-MS. LILBID spectra are showing the membrane protein complexes KcsA tetramer (**a**) and DgkA trimer (**c**). nESI spectra are showing KcsA tetramer (**b**) but no DgkA trimer (**d**). Complex concentrations were 10 μM in 100 mM ammonium acetate and 5× CMC DDM if not indicated differently

Figure 6a shows a LILBID spectrum with the tetrameric KcsA as the predominant species. The tetramer can be confirmed with nESI as well, even though even under optimized conditions (60 V) the tetramer appears as a minor species only (Figure 6b).

The expected DgkA trimer in DDM is the predominant species seen with LILBID, presented in Figure 6c (top).

nESI spectra of DgkA show monomers and dimers in DDM in Figure 6d (top). No trimeric state could be observed, which is in line with previous studies [36]. As Brij 35 is known to be an especially mild detergent we used it to substitute the DDM. nESI spectra showed no improvement. Only monomeric DgkA could be observed in the presence of Brij 35 (Figure 6d bottom).

The same sample investigated with LILBID showed DgkA trimers, albeit slightly higher charged if compared to the DDM solubilized spectra (Figure 6c bottom).

For all proteins, the predominant signals in LILBID correspond to the native oligomeric state of the membrane proteins EmrE (dimer), DgkA (trimer), and KcsA (tetramer). Out of the three tested membrane proteins, nESI could confirm the correct oligomeric state for EmrE and KcsA. The predominant signal in nESI was in all cases under all conditions the membrane protein monomer. The here-investigated membrane proteins consist mainly of transmembrane helixes with only small soluble parts. As the membrane part of the complexes are covered by the detergent micelles the proteins are only visible in the mass spectra if the micelles are removed by collisional activation. This can lead at the same time to complex dissociation, which we observed for the here investigated proteins. This is less of an issue for complexes consisting of membrane parts and large soluble parts, such as an ATPase [10], which can pick up the charges in the nESI process [3] In comparison the LILBID desorption process is not biased towards soluble proteins areas, which shows that LILBID is especially suited to investigate membrane protein complexes.

## Conclusion and Outlook

The here presented study shows a comparison between the native MS ionization techniques nESI and LILBID. We investigated the ion release, clean-up efficiency and dissociation options in instruments with both sources as well as tolerance towards additives and buffers for both soluble and membrane proteins. The limit of detection and sample consumption are equal. A directly noticeable difference are the protein charges in the mass spectra. As no active charging occurs during the ionization process in LILBID, proteins generally carry less charges than after ionization with nESI.

Both methods perform well for the analyzed water soluble protein complex, allowing to determine the correct oligomeric state.

Membrane proteins are generally more challenging to investigate with mass spectrometry.

While complexes consisting of membrane and soluble parts have been investigated successfully with nESI, [46] proteins consisting mostly of TMHs are challenging [47]. The droplets produced in the nESI spray shrink during the evaporation process, causing detergent concentration. Therefore, increased collisional clean-up is needed to remove a sufficient amount of detergents from the complex, which can cause unwanted dissociation of the membrane protein complex as well.

LILBID reveals reliably the intact membrane protein complexes as the predominant species and is therefore especially suited to investigate membrane proteins. The rapid protein complex release from solution into the gas phase effectively removes detergent from the complex, without dissociating it at the same time. This is essential for structural investigations of proteins with native MS.

Controlled dissociation of protein complexes can be of interest to reveal the constituting subunits or structural information. Both instruments/methods offer an option to transfer additional energy into the complex to achieve such a dissociation. The nESI process itself is generally not manipulated to trigger dissociation, but the gas phase ions can be submitted to a collision cell. There the ions collide with inert gas atoms (generally within a timeframe of milliseconds [33]), which in dependence of collision energy leads to CIU and CID, which removes typically one or two peripheral proteins [41].

The laser irradiation process of LILBID occurs on a much faster time scale (6 ns pulse length) [8]. This allows effective removal of detergent and gives the option of controlled complex dissociation into subcomplexes or all subunits. Accordingly, variation of the laser power in LILBID can reveal information on subunit composition and arrangement as well as different binding strength for different interfaces, as seen for Avidin. Ramping the LILBID laser energy showed the tetrameric Avidin disassembling via dimers into monomers, which is in line with the substructural arrangement of Avidin monomers in the quaternary structure. Information on the arrangement of Avidin (analogues) have been successfully investigated by using nESI combined with surface-induced dissociation (SID) instead of CID [40, 48].

SID is a short single ion release event on a time scale comparable to the LILBID laser energy transfer, which might explain why LILBID and SID dissociation patterns have more in common than CID.

An exciting feature is the comparably high tolerance of LILBID towards addition of salt and other additives, such as non-volatile buffers. The laser desorption mechanism does not include the droplet shrinking process, which leads to concentration of additives in the nESI process. Therefore, higher starting concentrations of additives can be tolerated, albeit they might lead to an increase in observed mass due to attachments. Nevertheless, even mass spectra afflicted with intensive peak broadening due to attachments, still allow for the unambiguous assignment of the low charged LILBID peak charge states. This can be an advantage as it enables even for large protein complexes the determination of the charge states and therewith oligomerization state, which can be difficult for nESI peak distributions if attachments cannot be removed sufficiently [46].

Up to now, it is noticeable that the mass resolution of LILBID spectra shown here is lower than in nESI spectra. The instrumental resolution of LILBID-TOF-MS could be improved by implementing an orthogonal time-of-flight (TOF) analyzer. The limits of achievable resolution with modern mass spectrometers are often due to incomplete desolvatization of the analyte. This state has not been reached for LILBID-MS, where instrumental improvements are still underway.

Another promising development project for the future will be the hyphenation of LILBID with ion-mobility (IM), as it is already successful with nESI. The low-charged LILBID ions would be a promising target for IM investigations as no distorting effect on the size and stability, as seen for highly charged nESI protein complexes [49], would be expected for LILBID ions.

The results from LILBID and nESI measuremenst show that both techniques can be used in a complementary manner. Depending on the question(s) to be answered for the investigated complex we use one or the combination of both instruments.

The presented work is an example of the ongoing development of native MS technology driven by the requirements of structural biology to broaden the applicability of MS based methods for protein complexes.

## Electronic supplementary material

ESM 1 (DOCX 2436 kb)
