# Supplementary material for: LILBID and nESI: Different Native Mass Spectrometry Techniques as Tools in Structural Biology
Source: J Am Soc Mass Spectrom. 2018 Sep 17;30(1):181–91. doi: 10.1007/s13361-018-2061-4 (PMC6318263; doi:10.1007/s13361-018-2061-4)
Supplement: Supplementary file 1 — (DOCX 2436 kb) [file 13361_2018_2061_MOESM1_ESM.docx]

LILBID and nESI: different native mass spectrometry techniques as tools in structural biology

Oliver Peetz^1§^, Nils Hellwig^1§^, Erik Henrich^2§^, Julija Mezhyrova^2^, Volker Dötsch^2^, Frank Bernhard^2^, Nina Morgner^1^*

^1^Institute of Physical and Theoretical Chemistry, J.W. Goethe-University, Frankfurt am Main, Germany

^2^Institute of Biophysical Chemistry, Centre for Biomolecular Magnetic Resonance, J.W. Goethe-University, Frankfurt am Main, Germany

^§^ *contributed equally*

Correspondence to: Nina Morgner; [morgner@chemie.uni-frankfurt.de](mailto:morgner@chemie.uni-frankfurt.de)

***Sample purification:***

The P-CF produced membrane protein precipitates were collected by centrifugation at 18,000 xg for 10 min followed by a washing step with buffer (20 mM Tris-HCl, pH.8.0 and 150 mM NaCl) and resolubilized for 1h at 30 °C in resuspension buffer (100 mM TRIS-HCl, pH 8.0, 100 mM NaCl, 2-3% DDM) with a volume corresponding to the reaction mixture. The resolubilization buffers contained 1 mM TPP for EmrE for stabilizing the dimer complex [23] and 1 mM TCEP for DgkA. After incubation the sample was again centrifuged at 18,000 xg to separate insoluble precipitates. Since the purification strategies varied slightly, the single workflows are presented separately.

Solubilized P-CF produced DgkA was diluted 1:3 with buffer D1 (100 mM Tris-HCl, pH 8.0, 100 mM NaCl, 1 mM TCEP, 0.1% DDM) without DDM and loaded onto an equilibrated Ni-NTA column. The flow through was re-applied 4 times to enhance binding. After binding, the column was washed with 5 column volumes (cv) buffer D1, 5 cv D1 with additional 200 mM NaCl and 20 mM imidazole and 5 cv D1 with additional 25 mM imidazole. The sample was eluted from the column with 4 cv of D1 supplemented with 300 mM imidazole. The elution was diluted 1:1 with D1 before concentration with 4 ml Amicon Ultra centrifugal filters with a molecular weight cut-off (MWCO) of 10 kDa by multiple centrifugation steps at 4,000 xg for 5 min. During centrifugation, the sample was diluted again to reduce the residual amount of imidazole below 5 mM.

Solubilized P-CF produced EmrE was diluted 1:3 with buffer E1 (100 mM Tris-HCl, pH 8.0, 100 mM NaCl, 1 mM TPP, 0.04% DDM) without DDM and loaded onto an equilibrated Ni-NTA column. The flow through was re-applied 4 times to enhance binding. After binding, the column was washed with 5 cv buffer E1, 5 cv E1 with the addition of 200 mM NaCl and 5 cv with the addition of 25 mM imidazole. The sample was eluted with 6 times 33% of the column volume of buffer E2 (100 mM Tris-HCl, pH 8.0, 100 mM NaCl, 1 mM TPP, 0.03% DDM, 300 mM imidazole). The protein containing fractions were collected separately without subsequent concentration.

After D-CF expression of DgkA the reaction mix was centrifuged at 18,000 xg for 10 min to separate insoluble components. The supernatant was diluted 1:1 with buffer D2 (100 mM Tris-HCl, pH 8.0, 100 mM NaCl, 1 mM TCEP, 0.1% Brij 35) and loaded onto an equilibrated Ni-NTA column. The flow through was re-applied 4 times to enhance binding. After binding, the column was washed with 5 cv buffer D2, 5 cv D2 with additional 200 mM NaCl and 20 mM imidazole and 5 cv D2 with additional 25 mM imidazole. The sample was eluted from the column with 4 cv of D2 supplemented with 300 mM imidazole. The elution was diluted 1:1 with D2 before concentration with 0.5 ml Amicon Ultra centrifugal filters with a MWCO of 10 kDa by multiple centrifugation steps at 11,000 xg for 10 min. During the centrifugation, the sample was diluted again to reduce the residual amount of imidazole below 5 mM.

L-CF reactions of KcsA were centrifuged at 18,000 xg for 10 min to separate insoluble components. The supernatant was diluted 1:1 with buffer K1 (100 mM Tris-HCl, pH 8.0, 100 mM NaCl) and loaded onto an equilibrated Strep-column. The flow through was re-applied 4 times to enhance binding. After binding, the column was washed with 10 cv K1. The sample was eluted from the column with 3 cv of K1 supplemented with the addition of 20 mM desthiobiotin. The elution was concentrated to ~1 ml filled into a Slide-a-Lyzer device from Thermo Scientific with a MWCO of 10 kDa and dialyzed against 2 l buffer K1 overnight. After dialysis, the sample was adjusted to 1% DDM by stepwise addition of a 10% DDM stock solution and incubated for 30 min to disrupt the nanodiscs. The sample was diluted 1:1 with buffer K1 and loaded onto an equilibrated Strep-column with buffer K1 supplemented with 0.05% DDM to separate StrepII-tagged KcsA from His_6_-tagged MSP1E3D1. The column was washed with 6 cv of buffer K1 with 0.05% DDM before elution with 10 times 33% of the cv with buffer K1 supplemented with 0.03% DDM and 20 mM desthiobiotin. The protein containing fractions were concentrated with 4 ml Amicon Ultra centrifugal filters with a MWCO of 10 kDa by multiple centrifugation steps at 4,000 xg for 5 min.

The proteins were buffer exchanged directly before MS measurement in the desired buffer environment using Zeba Micro Spin Desalting Columns (article number 89887) from Thermo Scientific. These columns are operating with a 7 kDa cut-off filter. For equilibration the desalting column was washed five times with the buffer at 1500 g for 1 minute and the final sample buffer exchange was done for 2 minutes at 1500 g.

3 µl of buffer exchanged sample were used for each measurement for both instruments and measured in parallel to avoid any possible sample ageing. All protein concentrations were controlled before and after buffer exchange by a Nanodrop spectrometer (NanoPhotomer NP80, IMPLEN, Munich, Germany). The molar absorption coefficients of all protein monomers are listed in Table 1.

The pH of all buffers was controlled and adjusted to the desired value by pH meter from METTLER TOLEDO (FE20, Giessen, Germany).

**Table S1:** Molare absorption coefficients and averaged mass of the proteins based on monomer.

| (membrane) protein | Molare absorption  coefficent [M^-1^cm^-1^] | Measured wavelength [nm] | Mass [g mol^-1^] |
| --- | --- | --- | --- |
| Avidin | 24280 | 280 | 15728 |
| DgkA | 20480 | 280 | 14285 |
| Emre | 35075 | 280 | 12999 |
| KcsA | 40450 | 280 | 21304 |

***LILBID settings:***

The voltage of the first (Repeller) and second lense was set to -4 kV in the Ion Source. The third lense is grounded. The Repeller was pulsed to - 6,6 kV for 370 µs after droplet irradiation. The puls was applied between 2-50 µs after the droplet irradiation (delayed extraction time). The einzel lenses were set between -2,0 kV up to -3,0 kV. The Reflectron was set to -7.2 kV and the TOF operates at 10^-6^ mbar. Post-acceleration was set to +17 kV at the MCP impact surface. Droplet production and laser irradiation was operating at 10 Hz.

***nESI settings and needle preparation:***

Offline needles (GC100TF-10, Harvard Apparatus Limited, Cambridge, UK) were produced in house using a needle puller (P-1000, Novata, CA, USA) and subsequently coated with platin/palladium in a sputter coater (Q 150R S, Leica Microsystems, Wetzlar, Germany). Pt/Pd is a higher resistance coating in contrast the more commonly used Au coating. Therefore, higher capillar voltage have to be applied. The voltage was adjusted to the lowest value resulting in a stable spray.

The Synapt G2-S instrument was operated in the positive nanoflow ESI mode. All critical instrument voltages, flows and pressures are as follows: capillary voltage 2.0 to 2.5 kV for higher resistance coating (Pt/Pd); sample cone 150 V; source block temperature 30° C; source offset 150 V; trap gas flow 7.00 ml min^-1^; helium cell gas flow 180 ml min^-1^; IMS argon gas flow 90 ml min^-1^; static offset 180 V; trap collision energy 10 V to 200 V; transfer collision energy 10 V; trap entrance 0.0 V; trap bias 3.2 V; trap DC -2.0 V; trap exit 1.0 V; trap wave velocity 300 m s^-1^; trap wave height 1.2 V; IMS entrance -20 V; IMS helium cell DC 1.0 V; IMS helium exit -20 V; IMS bias 2.0 V; IMS DC exit 20 V; transfer wave velocity 247 m s^-1^; transfer wave height 1.0 V; transfer DC entrance 5.0 V, transfer DC exit 15.0 V; IMS wave velocity 300 m s^-1^; IMS wave height 1.0 V; mobility trapping time release 500 µs; mobility trap wave height 1.2 V; mobility extract height 0.0 V; backing pressure 3.88 mbar, source 7,85e^-3^ mbar, trap pressure 2.21e^-2^ mbar , helium cell pressure 3.37e^-4^ mbar, IMS cell pressure 4.92e^-4^ mbar; transfer cell pressure 2.25e^-2^ mbar, TOF pressure 1.41e^-6^ mbar.

**Limit of Detection (LOD) of LILBID-MS and nESI-MS**

**
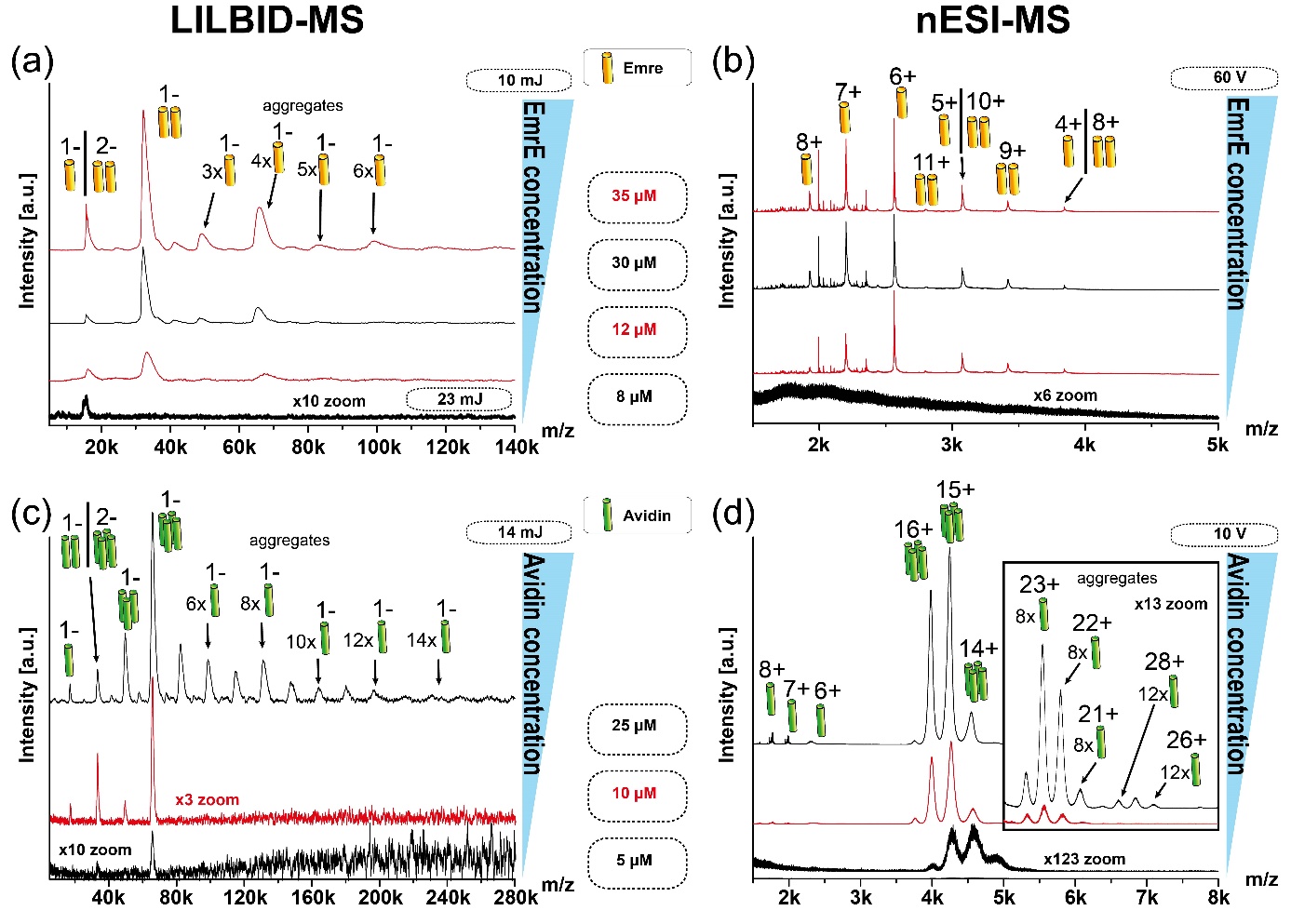
**

**Figure S1.** Concentration screening to monitor the limit of detection and aggregate formation of EmrE (**a**-**b**) and Avidin (**c**-**d**). The limit of detection for the EmrE complex is between 12 µM and 8 µM for both MS techniques (**a**-**b**) and around 5 µM for the Avdin complex (**c**-**d**), showing a similar LOD for both instruments. EmrE aggregates from 3mers to 5/6mers start to appear in the LILBID spectra from 12 µM protein concentration. (**a**-**b**). Avidin aggregates up to 14mers can be found with LILBID for the 25 µM protein sample. nESI spectra show Avidin aggregates already in the 10 µM protein samples. Suprisingly, only aggregates from two Avidin complexes (8mer) or 3 avidin complexes (12mer) could be found in nESI (**d**), showing different aggregation results due to different ion release mechanism

Supporting information on buffer tolerance of LILBID and nESI


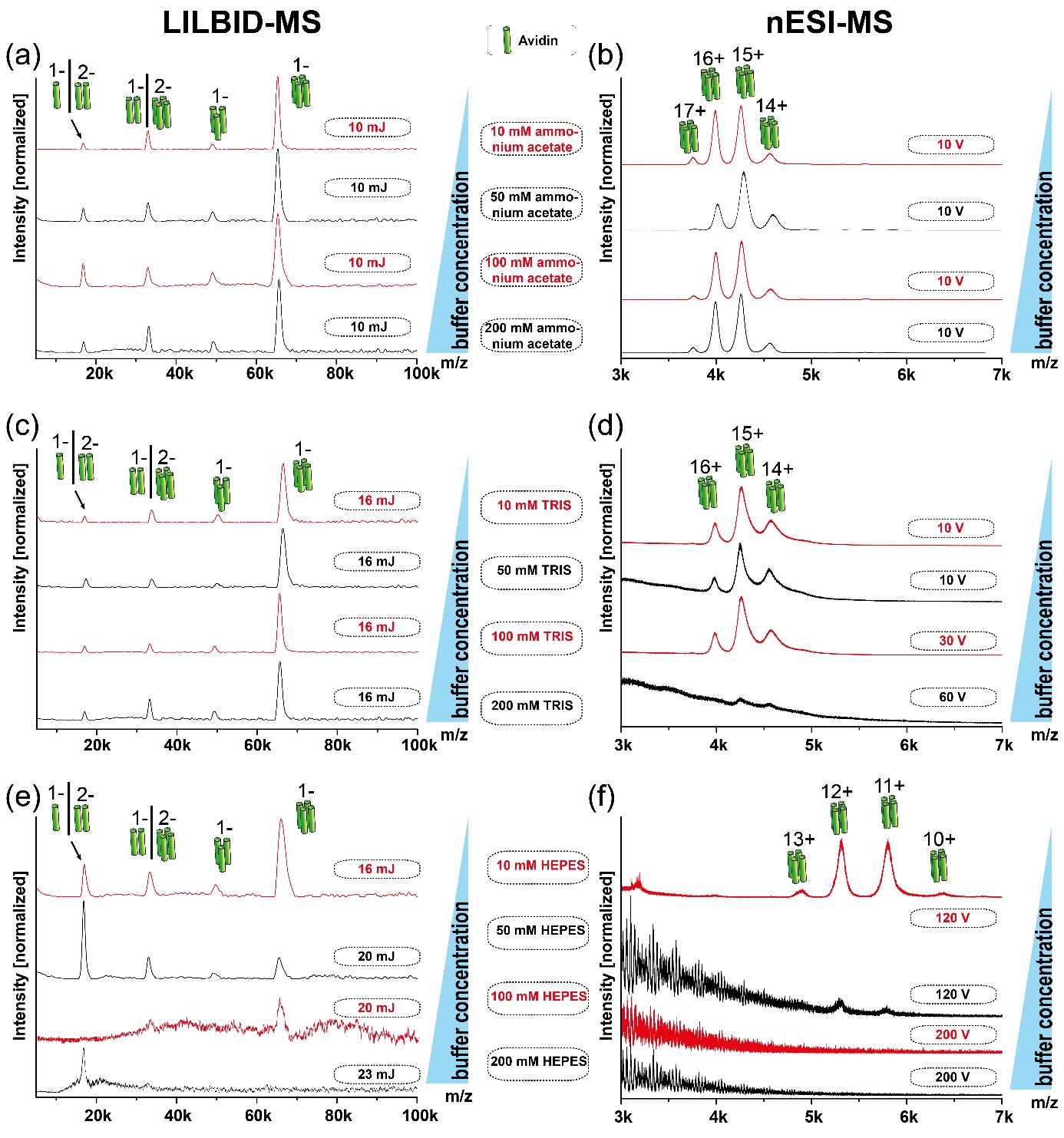


**Figure S2.** Influcence of the concentrations of the buffers ammonium acetae (**a**-**b**), TRIS (**c**-**d**) and HEPES (**e**-**f**) on Avidin spectra quality. The Avidin tetramer in ammonium acetate buffers ranging from 10 mM to 200 mM is clearly detectable with LILBID (**a**) and nESI (**b**) at 10 mJ and 10 V CID respectively. Non-volatile buffers like TRIS can be used up to 200 mM with LILBID, even though more laser power is needed to release the Avidin tetramer from the droplet in LILBID (**c**). Avidin tetramer can be assigned clearly up to 100 mM TRIS using nESI (**d**). As with LILBID, harsher instrumental settings are needed to release the complex, as indicated. HEPES buffer is more challenging, limiting the complex detection at 100 mM HEPES for LILBID. At 200 mM HEPES the detection of the monomer is possible, but only at conditions which dissociate the tetramer. (**e**). The HEPES concentration limit in nESI lies between 10 mM and 50 mM HEPES (**f**). Both MS techniques needed harsh settings to monitor the avidin complex

**
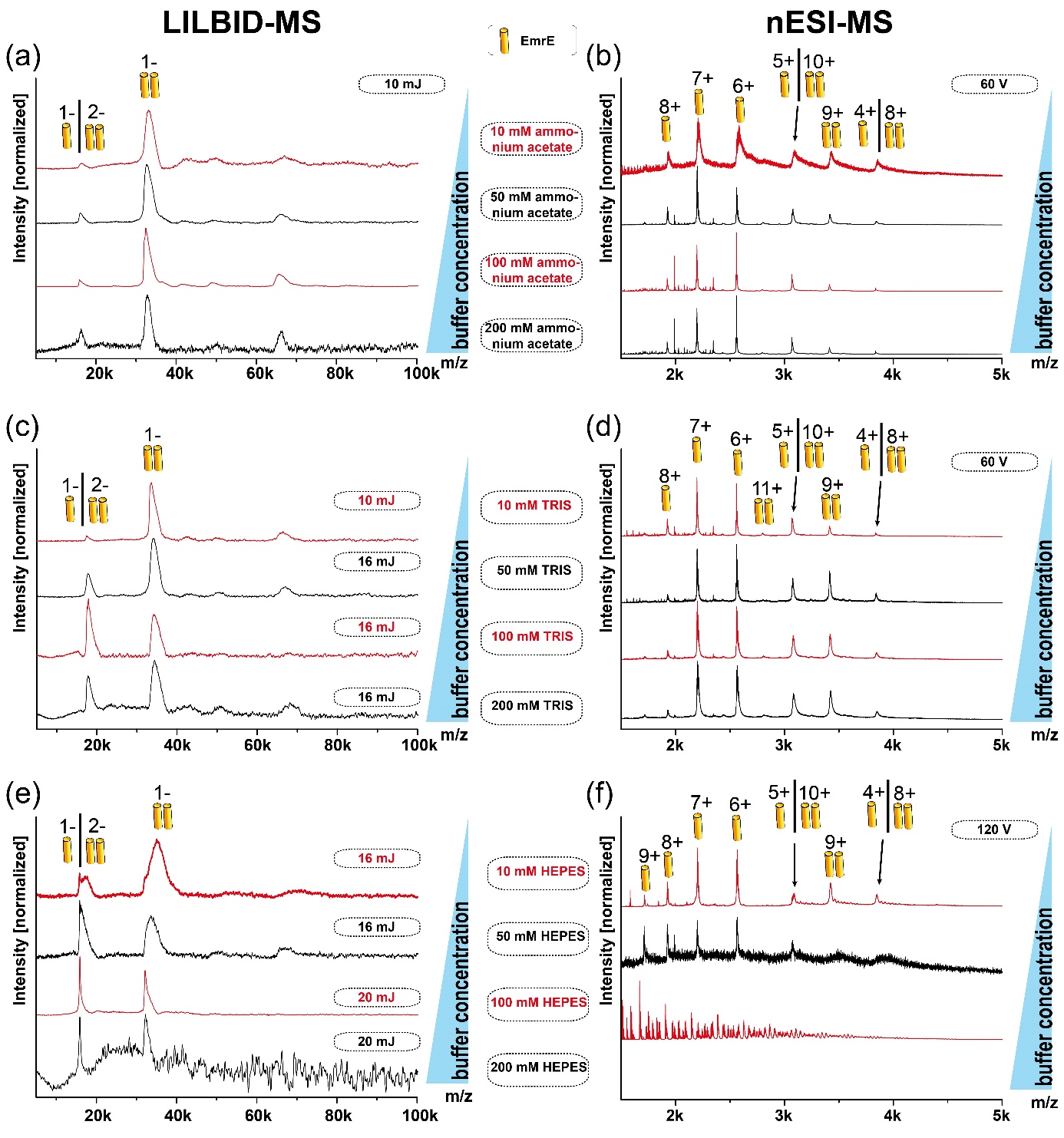
 Figure S3**. Influence of the buffers ammonium acetate (**a-b**), TRIS (**c-d**) and HEPES (**e-f**) on EmrE spectra quality. The EmrE spectra are resolved in up to 200 mM ammonium acetate with LILBID (**a**) as well as in nESI (**b**). Increased concentrations of the volatile buffer ammonium acetate even leads to improved spectral resolution for nESI. This effect cannot be seen for LILBID spectra. EmrE dimer can be seen in TRIS up to 200 mM with LILBID (**c**) and nESI (**d**). EmrE dimer can be monitored in up to 200 mM HEPES by LILBID, albeit at reduced spectra quality (**e**). In contrast, the dimer could be detected clearly with nESI for the sample in 10 mM HEPES, while the spectra at 50mM HEPES merely hints at the existence of a dimer (**f**)

Supporting information on influence of salt, glycerol and detergent concentration on LILBID and nESI spectra


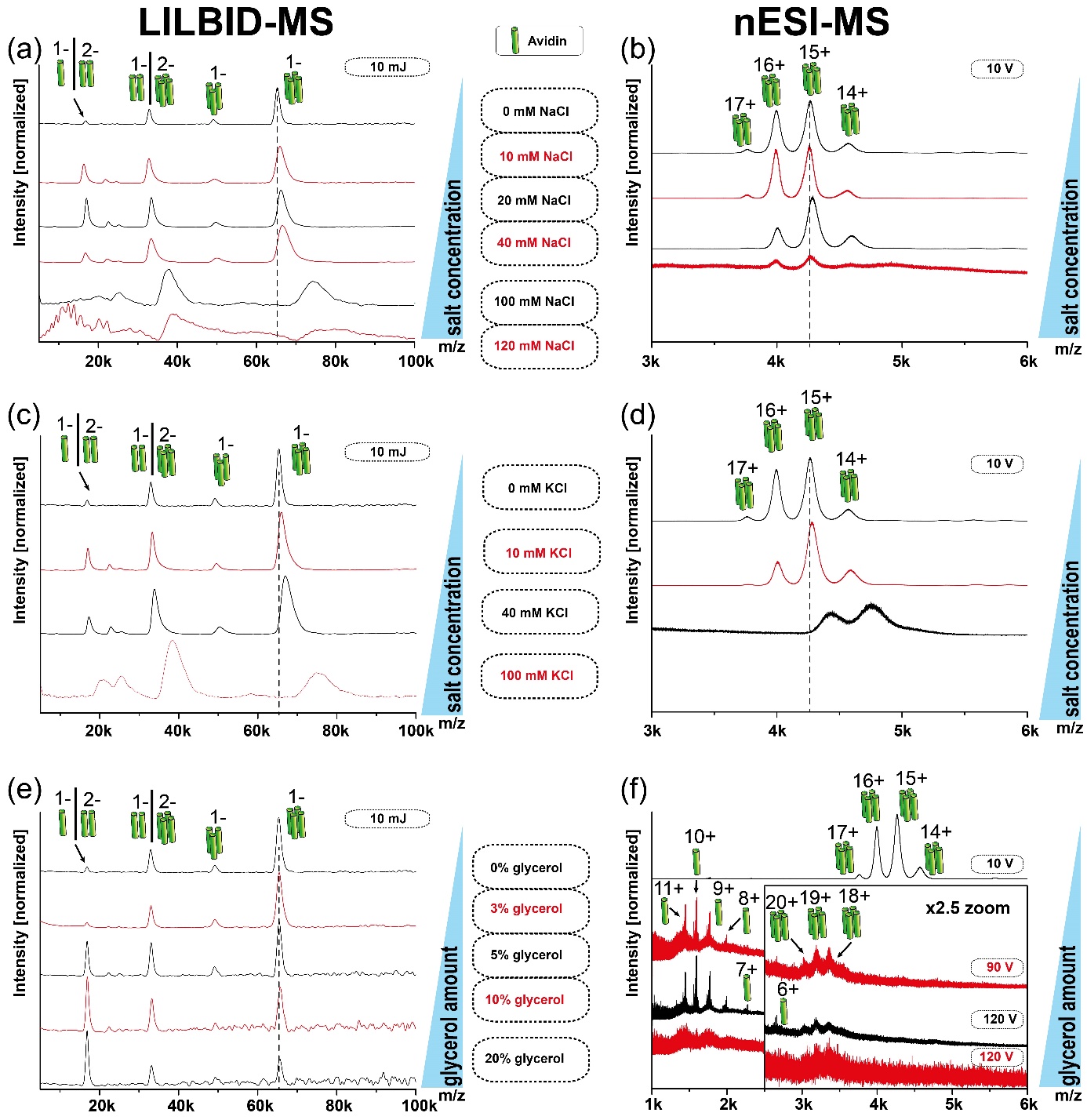


**Figure S4.** Influcence of salt (**a-d**) and glycerol (**e-f**) on Avidin**.** The Avidin tetramer is clearly detectable in LILBID up to 100 mM NaCl (**a**) or 100 mM KCl (**c**) at 10 mJ laser power (**a**). In contrast, in nESI the Avidin tetramer was only detectable up to 40 mM NaCl (**c**) and 40 mM KCl (**d**), investigated at 10 V CID. Increasing salt concentration leads to increase of the detected complex mass with LILBID and nESI, due to salt adducts, as indicated by dotted lines. 40 mM KCl shifts the mass of avidin tetramer in nESI and LILBID spectra about 2.4 kDa and 1.8  kDa respectivley. 100 mM NaCl or 100 mM KCl cause an increase of the tetramer masses in LILBID of about 9.1 kDa and 9.8 kDa respectivley. Avidin tetramer in solution containing up to 20% glycerol is still detectable with LILBID at 10 mJ (**e**). With increasing glycerol amount the tetramer stability decreases showing increased dimer and monomer signal from 5% glycerol. For nESI measurements the CID voltage has to be increased steadily with increasing glycerol amount, indicating a minimum CE voltage is required as indicated to obtain protein signals (**f**). Glycerol is a known supercharging agent in nESI, shown by an averaged increase of 3 protons for the avidin tetramer. At 10% glycerol, the LOD for avidin tetramer is reached in nESI


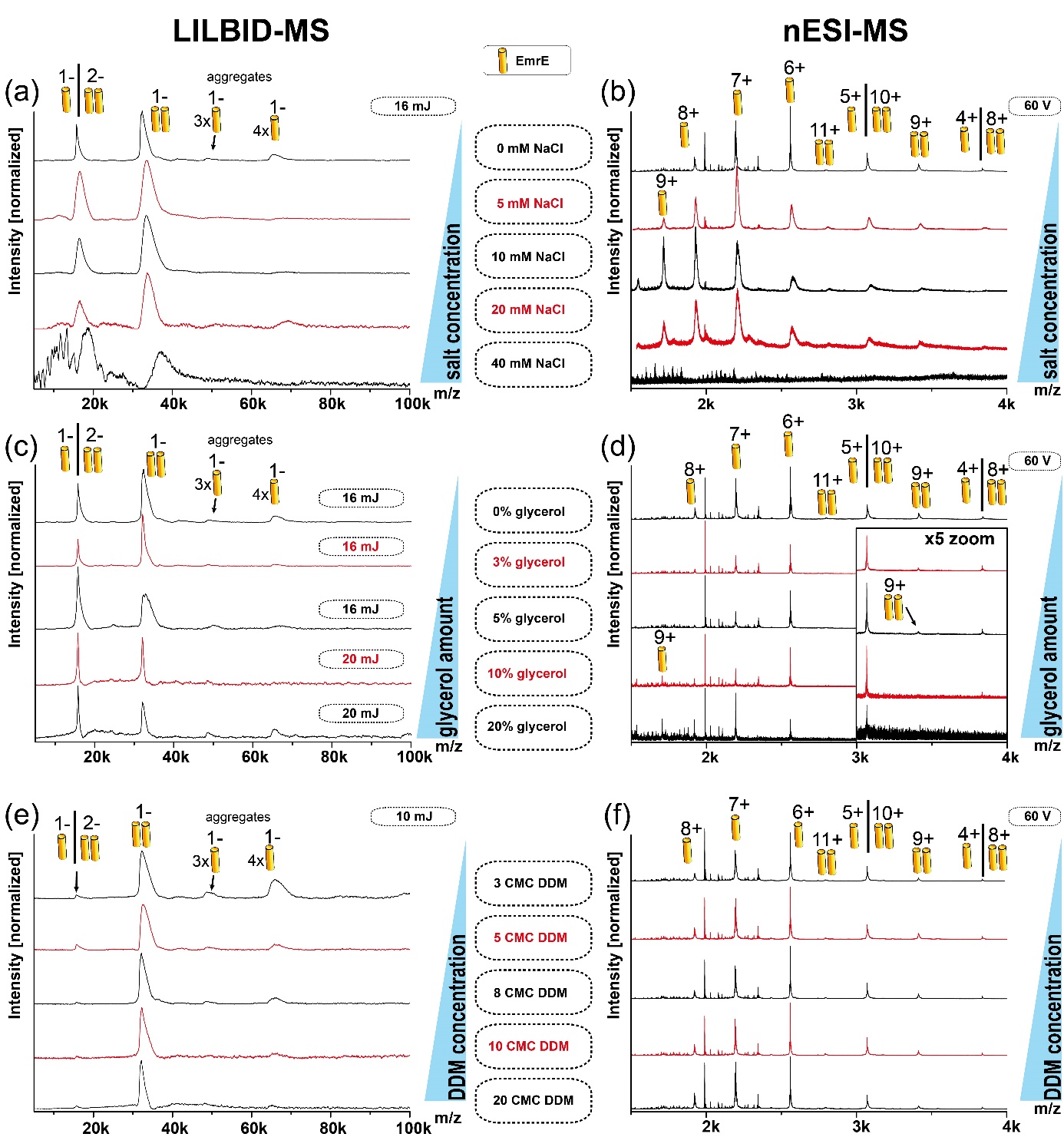


**Figure S5.** Influcence of salt (**a-b**), glycerol (**c-d**) and detergent DDM concentration (**e-f**) on EmrE. EmrE dimer is visible up to 40 mM NaCl in LILBID (**a**) and up to 20 mM NaCl in nESI (**b**). Increasing amount of Glycerol destabilizies the EmrE dimer seen for addition from 5% glycerol in LILBID spectra (**c**) as well as for nESI (**d**). At higher glycerol concentrations no dimer is visible with nESI, as seen in the zoom in region (**d**). The EmrE dimer is detectable in DDM ranging from 3 x CMC DDM up to 20 x CMC in LILBID at 10 mJ (**e**) and nESI at 60 V CID (**f**). The spectra of 30 µM EmrE dimer complex recorded with either glycerol or salt contained 5x CMC DDM at pH 6.8 in 100 mM ammonium acetate
